# Euthanasia and assisted suicide: An in-depth review of relevant historical aspects

**DOI:** 10.1016/j.amsu.2022.103380

**Published:** 2022-02-11

**Authors:** Yelson Alejandro Picón-Jaimes, Ivan David Lozada-Martinez, Javier Esteban Orozco-Chinome, Lina María Montaña-Gómez, María Paz Bolaño-Romero, Luis Rafael Moscote-Salazar, Tariq Janjua, Sabrina Rahman

**Affiliations:** a Medical and Surgical Research Center, Future Surgeons Chapter, Colombian Surgery Association, Bogotá, Colombia; b Grupo Prometheus y Biomedicina Aplicada a las Ciencias Clínicas, School of Medicine, Universidad de Cartagena, Cartagena, Colombia; c Department of Medicine, RedSalud, Santiago de Chile, Chile; d Department of Medicine, Keralty Salud, Bogotá, Colombia; e Colombian Clinical Research Group in Neurocritical Care, Latin American Council of Neurocritical Care, Bogotá, Colombia; f Department of Intensive Care, Regions Hospital, Minnesota, USA; g Independent University, Dhaka, Bangladesh

**Keywords:** Euthanasia, Assisted suicide, Terminal care, Bioethical issues, History of medicine

## Abstract

End-of-life care is an increasingly relevant topic due to advances in biomedical research and the establishment of new disciplines in evidence-based medicine and bioethics. Euthanasia and assisted suicide are two terms widely discussed in medicine, which cause displeasure on many occasions and cause relief on others. The evolution of these terms and the events associated with their study have allowed the evaluation of cases that have established useful definitions for the legal regulation of palliative care and public policies in the different health systems. However, there are still many aspects to be elucidated and defined. Based on the above, this review aimed to compile relevant historical aspects on the evolution of euthanasia and assisted suicide, which will allow understanding the use and research of these terms.

## Highlights

• The history of euthanasia and assisted suicide has been traumatic.
• The church and research have been decisive in the definition of euthanasia.
• The legal framework on the use of euthanasia and assisted suicide has been strengthened.

## 1 Introduction

Euthanasia and assisted suicide are two topics discussed throughout history, mainly because they fall within the scope of life as a human right, which has been universally defended for many years [1]. However, the mean of the word euthanasia as good death generates conflicts at social, moral, and ethical levels. Mainly because death is a loss, it is difficult to understand it as something positive and; additionally, several historical events such as the Nazi experiments related the term euthanasia more to murder than to a kind and compassionate act [1]. More current texts mention that euthanasia is the process in which, through the use or abstention of clinical measures, the death of a patient in an incurable or terminal condition can be hastened to avoid excessive suffering [2].

The difference between euthanasia and assisted suicide is that in the latter, the patient takes the final action; however, both practices can be combined in the term assisted death [2]. At present, several countries authorize assisted death, including Holland, Luxembourg, and Canada [3]. Belgium and Colombia have regulations that decriminalize only euthanasia; other places where assisted suicide is legal are Switzerland and five states of the United America states, specifically Oregon, Vermont, Washington, California, and Montana [2,3]. Spain recently joined the list of countries that have legislated on euthanasia through the organic law March 2021 of March 24 that regulates euthanasia in that state in both public and private institutions [4]. The fact that more and more countries were joining the legislation on euthanasia and assisted suicide has brought to light the opinion of thinkers, politicians, philosophers, and physicians. Several nations have initiated discussions on the matter in their governmental systems. Latin America is trying to advance powerfully in this medical-philosophical field. Currently, in Chile, the “Muerte digna y cuidados paliativos” law, which seeks to regulate the issue of euthanasia and assisted suicide in the country, is being debated in Congress [5].

It is essential to know the point of view of physicians on euthanasia and assisted suicide, especially taking into account that these professionals who provide care and accompany patients during this moment, which, if approved, would involve the medical community in both public and private health systems. Although it seems easy to think that physicians have a position in favor of the act of euthanasia because they are in direct and continuous contact with end-of-life situations, such as palliative care, terminally ill, and critically ill patients. It is important to remember that the Hippocratic medical oaths taken at the time of graduation of professionals are mostly categorical in mentioning the rejection of euthanasia and assisted suicide [6]. Furthermore, it is also important to note that many of the oldest universities in the Western world originated through the Catholic Church; and just this creed condemns the practice of euthanasia and continues to condemn it to this day. This situation generates that many medical students in these schools have behaviors based on humanist principles under the protection of faith and religion and therefore reject the possibility of euthanasia [7,8].

The relevance of the topic and the extensive discussion that it has had in recent months due to the COVID-19 pandemic added to the particular interest of bioethics in this topic and the need to know the point of view of doctors and other health professionals on euthanasia and assisted suicide.

## 2 Origin and meaning of the term euthanasia

The word euthanasia derives from the Greek word “eu” which means good, and the word “thanatos” which means death; therefore, the etymological meaning of this word is “good death”. Over time the evolution of the meaning has varied; even as we will see below was considered a form of eradication of people categorized under the designation of leading a less dignified life. Assisted suicide is a condition in which the patient is the one who carries out the action that ends his life through the ingestion of a lethal drug but has been dispensed in the context of health care and therefore called assisted. This care is provided by a physician trained in the area. However, it requires the prior coordination of a multidisciplinary team and even the assessment by an ethics committee to determine that the patient is exercising full autonomy, free from coercion by the situation he/she is living and free from the fatalistic desires of a psychiatric illness [9]. In a more literary sense, the word euthanasia meaning of “giving death to a person who freely requests it in order to free himself from suffering that is irreversible and that the person himself considers intolerable” [9].

Some authors go deeper into the definition and consider that for the meaning of euthanasia, are necessary to consider elements that are essential in the word itself; such as the fact that it is an act that seeks to provoke death and that carried out to eliminate the suffering in the person who is dying. Other elements with a secondary character in the definition are the patient's consent (which must be granted respecting autonomy and freedom in the positive and negative sense; that means the fact must be not be coerced in any way). Another element is the terminal nature of the disease, with an irreversible outcome that generates precariousness and a loss of dignity. The third secondary element is the absence of pain of the death through the use of drugs such as high-potency analgesics, including opioids, high-potency muscle relaxants, and even anesthetic drugs. Finally, the last element is the health context in which the action is performed (essential in some legislations to be considered euthanasia) [10]. According to the World Health Organization, the union of these two components is the current definition of euthanasia, which describes as “the action performed by a person to cause the painless death of another subject, or not preventing death in case of terminal illness or irreversible coma. Furthermore, with the explicit condition that the patient must be suffering physical, emotional, or spiritual and that affliction is uncontrollable with conventional measures such as medical treatments, analgesics, among others; then the objective of euthanasia is to alleviate this suffering” [11]. Unfortunately, the term euthanasia has been misused over the years, and other practices have been named with this word. An example of this situation occurred during the Nazi tyranny when the word euthanasia concerned the murder of people with disabilities, mental disorders, low social status, or gay people. At that time, euthanasia was even a simultaneous practice to the Jewish genocide [11].

Not only has the term been misused; also exists an enormous variability of terms to refer to euthanasia. For example, the laws created to regulate euthanasia have different names around the world; in the Netherlands (Holland), the law that regulates this practice is known as the law of termination of life; in Belgium, it is called euthanasia law, in France, it is called euthanasia law too. In Oregon (USA), it is called the death with dignity act; in California, it is the end of life option act. In Canada is called the medical assistance in dying act. Victoria (Australia) is the voluntary assisted dying bill, but all these denominations refer to the already well-known term euthanasia [11].

## 3 Evolution of euthanasia and assisted suicide: digging into historical events

To understand the evolution and relevance of these concepts should analyze the history of euthanasia and assisted suicide; from the emergence of the term, going through its first manifestations in antiquity; mentioning the conceptions of great thinkers such as Plato and Hippocrates; going through the role of the Catholic Church; mainly in the Middle Age, where following the thought of St. Thomas Aquinas, self-induced death or death contemplated by own will, was condemned. Later, with the renaissance age and the resurgence of science, technology, and the arts, the term euthanasia made a transition to a form similar to what we know today from thinkers such as Thomas More and Francis Bacon. Finally, the first signs of eugenics were known in London, Sweden, Germany, and the United States in the twentieth century. There was a relationship with the term euthanasia that was later used interchangeably, especially in the Nazi regime, to denote a form of systemic murder that sought to eradicate those who were not worthy of living a life.

Since the sixties, with emblematic cases, the path towards the decriminalization of euthanasia began in some countries, especially concerning the cessation of extreme support measures in cases of irreversible illness or a terminal condition. The practice has progressed to the appearance of laws on euthanasia in several countries.

## 4 Euthanasia and assisted suicide in ancient times

In book III of Plato's “The Republic”, the author stated that those who live their lives amidst illnesses and medicines or who were not physically healthy should be left to die; implying that it was thought that people in these conditions suffered so much that their quality of life diminished, which seemed understandable to these thinkers. However, other authors such as Hippocrates and his famous Hippocratic oath sought the protection of the patient's life through medicine, especially in vulnerable health conditions prone to fatal outcomes. This Hippocratic oath is the same oath that permeates our times and constitutes an argument among those who mark their position against euthanasia and assisted suicide [12,13].

Other texts that collect thoughts of Socrates and his disciple Plato point out that it was possible and well understood to think of ceasing to live in the face of a severe illness; to consider death to avoid a long and torturous agony. This fact is compatible with the conception of current euthanasia since this is the end of this health care procedure [13].

In The Republic, the text by Plato, the physician Heroditus is also condemned for inventing a way to prolong death and over manage the symptoms of serious illnesses, which is currently known as distanasia or excessive treatment prolongs life. This kind of excessive treatment prolongs the sick person's suffering, even leading him to maintain biological signs present but in a state of alienation and absolute dependence on medical equipment such as ventilators and artificial feeding [13]. However, the strongest indication that Euthanasic suicide was encouraged in Greece lies in other thinkers such as the Pythagoreans, Aristotelians, and Epicureans who strongly condemned this practice, which suggests that it was carried out repeatedly as a method and was therefore condemned by these thinkers [12,13]. According to stoicism, the pain that exceeded the limits of what was humanly bearable was one of the causes for which the wise man separates himself from life. Referring to one of the nuances that euthanasia touches today, that is, at a point of elevated suffering, the dignity and essence of the person are lost, persisting only the biological part but in the absence of the person's well-being as a being. In this sense, Lucius Seneca said that a person should not love life too much or hate it; but that person should have a middle ground and end their life when they ceased to perceive life as a good, worthy, and longed-for event [1,12].

During the Roman Empire and in the territories under its rule, it was believed that the terminally ill who commit suicide had sufficient reasons to do so; so since suicide caused by impatience and lack of resolution to pain or illness was accepted, when there was no access to medicines. In addition, there was little development in medicine during that time, and many of the sick died without treatment [12]. This situation changed later with the emergence of the Catholic church; in this age, who attempted against own life, was deprived of burial in the ground. Saint Augustine said that the suicide was an abominable and detestable act; from 693 AD, anyone who attempted against his physical integrity was excommunicated. Rejecting to the individuals and their lineage, depriving them of the possibility of attending the funeral and even expelled from cities and stripped of the properties they owned [12,13].

### 4.1 Euthanasia and assisted suicide in the Middle Age

During the Middle Age, Catholicism governed the sciences, arts, and medicine; the sciences fell asleep. Due to this solid religious tendency and the persistence of Augustinian thought, suicide was not well seen. It was not allowed to administer a lethal substance to a person to end the suffering of a severe or terminal illness [9,12]. People who took their own lives at this time could not be buried “Christianly”; therefore, they did not have access to a funeral, nor to the accompaniment of their family in a religious rite. Physical suffering and pain were then seen as a path to glorification. Suffering was extolled as the form that god purified the sin, similar to the suffering that Jesus endured during his Calvary days. However, a contrary situation was experienced in battles; a sort of short dagger-like weapon was often used to finish off badly wounded enemies and thus reduce their suffering, thus depriving them of the possibility of healing and was called “mercy killing” [12].

## 5 Euthanasia in renaissance

With the awakening of science and philosophy, ancient philosophers' thoughts took up again, giving priority to man, the world, and nature, thus promoting medical and scientific development. In their discourse, Thomas More and Francis Bacon refer to euthanasia; however, they give a eugenic sense to the concept of euthanasia, similar to that professed in the book of Plato's Republic. It is precise with these phylosophers that the term euthanasia got its current focus, referring to the acceleration of the death of a seriously ill person who has no possibility of recovery [12]. In other words, it was during this period that euthanasia acquired its current meaning, and death began to be considered the last act of life. Therefore, it was necessary to help the dying person with all available resources to achieve a dignified death without suffering, closing the cycle of life that ends with death [13,14].

In his work titled “Utopia”, Thomas More affirmed that in the ideal nation should be given the necessary and supportive care to the dying. Furthermore, in case of extraordinary suffering, it can be recommended to end the suffering, but only if the patient agrees, through deprivation of food or with the administration of a lethal drug; this procedure must be known to the affected person and with the due permission of authorities and priests [12,13]. Later, in the 17th century, the theologian Johann Andreae, in his utopia “Christianopolis”, contradicts the arguments of Bacon and Moro, defending the right of the seriously ill and incurably ill to continue living, even if they are disturbed and alienated, advocating for the care based on support and indulgence [15,16]. Similarly, many physicians rejected the concepts of Plato, Moro, and Bacon. Instead, they focused on opposing euthanasia, most notably in the nineteenth century. For example, the physician Christoph Hufeland mentioned that the doctor's job was only to preserve life, whether it was a fate or a misfortune, or whether it was worth living [16].

### 5.1 Euthanasia in the 20th century

Before considering the relevant aspects of euthanasia in the 20th century, it is vital to highlight the manuscript by Licata et al. [17], which narrates two episodes of euthanasia in the 19th century. The first one happened in Sicily (Italy) in 1860, during the battle of Calatafimi, where two soldiers were in constant suffering, one because he had a serious leg fracture with gangrene, and the other with a gunshot wound. The two soldiers begged to be allowed to die, and how they were in a precarious place without medical supplies, they gave them an opium pill, which calmed them until they died [17]. The second episode reported by Licata et al. [17] was witnessed by a Swedish doctor named Alex Munthe; who evidenced the pain of many patients in a Parisian hospital. So he decided to start administering morphine to help people who had been seriously injured by wolves and had a poor prognosis; therefore, the purpose of opioid use was analgesia while death was occurring.

It is also important to highlight the manuscript entitled “Euthanasia” by S. Williams published in 1873 in “Popular Science Monthly”, a journal that published texts by Darwin, Edison, Pasteur, and Beecher. This text included the report for the active euthanasia of seriously ill patients without a cure, in which the physicians were advised to administer chloroform to these patients or another anesthetic agent to reduce the level of consciousness of the subject and speed up their death in a painless manner [16].

Understanding that euthanasia was already reported in the nineteenth century, years after, specifically in 1900, the influence of eugenics, utilitarianism, social Darwinism, and the new currents of thought in England and Germany; it began in various parts around the world, projects that considered the active termination of life, thus giving rise to euthanasia societies in which there were discussions between philosophers, theologians, lawyers, and medical doctors. Those societies discussed diverse cases, such as the tuberculous patient Roland Gerkan, who was considered unfit and therefore a candidate to be released from the world [16]. The scarcity of resources, famine, and wars were reasons to promote euthanasia as a form of elimination of subjects considered weak or unfit, as argued in texts such as Ernst Haeckel's. However, opponents to the practice, such as Binding and Hoche, defended the principle of free will in 1920 [16].

### 5.2 Euthanasia in the time of the Nazis

As mentioned above, the term euthanasia was misused during this period; approximately 275,000 subjects (as reported at the Nuremberg International Military Tribunal 1945–1946), who had some degree of physical or mental disability, were killed during Adolf Hitler's Euthanasia program [13]. However, the Nazis were not the first to practice a form of eugenics under the name of euthanasia, since the early 1900s in London had already begun the sterilization of the rejected, such as the blind, deaf, mentally retarded, people with epilepsy, criminals, and rapists. This practice spread to different countries like Sweden and the United States [13,16].

For the Nazis, euthanasia represented the systematic murder of those whose lives were unworthy of living [13]. The name given to this doctrine was “Aktion T4”. At first and by law, from 1939, the hospitals were obliged to account for all disabled newborns, which led to the execution of more than 5000 newborns utilizing food deprivation or lethal injection [12,18].

A year before that law, in 1938, one of the first known cases of euthanasia in children arose in Germany. That history called the story of child K, in which it was the father of the minor who asked Hitler in writing for euthanasia for his son because the child had a severe mental disability and critical morphic disorders. Hitler gave his consent to carry out the procedure on child K, and thus the program began to spread throughout the Aleman territory. Since then, physicians and nurses had been in charge of reporting the newborns with alterations, arising the “Kinderfachabteilugen” for the internment of children who would be sentenced to death after a committee's decision [12,18,19]. A list of diseases and conditions that were considered undesirable to be transmitted to Hitler's superior Aryan race was determined; thus, any child with idiocy, mongolism, blindness, deafness, hydrocephalus, paralysis, and spinal, head, and hip malformations were eligible for euthanasia [19].

Subsequently, the program was extended to adults with chronic illness, so those people were selected and transported by T4 personnel to psychiatric sanatoriums strategically located far away. There, the ill patients received the injection of barbiturate overdoses, and carbon monoxide poisoning was tested as a method of elimination, surging the widely known gas chamber of the concentration camp extermination; this situation occurred before 1940 [12,19]. Again, physicians and nurses were the ones who designated to the patients to receive those procedures; in this case, these health professionals supported Nazi exterminations. They took the patients to the sanatoriums, where psychiatrists evaluated them and designated with red color if they should die and with a blue color if they were allowed to live (this form of selection was similar in children) [12,13,19]. In this case, the pathologies considered as criteria for death were those generating disability such as schizophrenia, paralysis, syphilis with sequelae, epilepsy, chorea, patients with chronic diseases with many recent treatments, subjects of non-German origin and individuals of mixed blood [19]. Once in the sanatoriums, they were informed that they would undergo a physical evaluation and take a shower to disinfect themselves; instead, they were killed in gas chambers [12,13]. Despite the church's action in 1941 against Nazis and after achieving suspension of the Aktion T4 project; the Nazi supporters kept the practices secretly, resuming them in 1942, with the difference that the victims were killed by lethal injection, by an overdose of drugs, or left to starve to death, instead of the use of gas chambers. This new modified form of euthanasia, which did not include gas chambers, became known as “savage euthanasia” [12,13,19].

### 5.3 Euthanasia since the 1960s

In September 1945, trials began for crimes perpetrated by Nazi supporters; the victorious Allied forces conducted these trials at the end of the war. During these tribunals, cases of human experimentation were identified and the public exposure of the Nazi euthanasia program. After the Nuremberg trials and the abolition of Nazi experiments, a series of seven documents emerged, among which the Nuremberg code containing the ten basic principles for human research stood out [20,21].

After these judgments, biotechnology was accelerated, with the apparition of new techniques to intervene in the health-disease process. Additionally, the increase in life expectancy and the appearance of diseases that chronically compromise the state of health of people generated a change in the conception of the critically ill patient and the terminal state of life [20,21]. Cases such as Karen Ann Quinlan brought to the forefront the issue of euthanasia and precisely the control of extreme treatment measures. Karen, a young American woman, was left in a vegetative state due to severe neurological damage following alcohol and barbiturate intoxication. After six months in that state and under the guardianship of a Catholic priest, Karen's parents requested the removal of the artificial respirator, arguing that in her state of consciousness prior to the incident, she had stated that she disagreed with artificially maintaining life in comatose patients. The hospital refused to remove the ventilator, arguing the legal issues for the date, and the parents went to court, which in the first instance granted the hospital the right. Nevertheless, the New Jersey Supreme Court granted Karen Ann's right to die in peace and dignity. Despite the withdrawal of the artificial respirator, he continued to live until 1985, when he finally died [[21], [22], [23]].

Another important case was Paul Brophy, which also occurred in the United States. Paul was a firefighter in Massachusetts and went into a deep coma due to the rupture of a basilar artery aneurysm; initially, his family advocated for support measures but later requested the hospital to disconnect these means to allow death, as Paul had indicated when he was still conscious. The hospital refused to carry out this procedure, so the family went to court, where the removal of the support measures (gastrostomy) was initially denied. Hence, the family went to the state supreme court, achieving the transfer of Paul to another medical center where the gastrostomy was removed, leading to his death within a few days [23].

The case of Arthur Koestler, an influential English writer and activist diagnosed with Parkinson's disease and later with leukemia, who served as vice-president of the voluntary euthanasia society (Exit) and wrote a manual book with practical advice for euthanasia called “Guide to Self-Liberation”. He stood out because he applied one of his advice and ingested an overdose of barbiturates, causing his self-death. According to his writings, Koestler was not afraid of death but of the painful process of dying [23]. In this sense, it was a relevant case because it involved someone who held an important position in an association that advocated euthanasia, in addition to being the author of several works, which made him a recognized public figure [23].

Baby Doe was a case that also occurred in the United States; it was a small child with Down syndrome who had a tracheoesophageal fistula and esophageal atresia; in this case, surgery was necessary. On the advice of the obstetrician, the parents did not allow surgery, so the hospital managers took the case before a judge who ruled that parents could decide to perform or not the surgery. The case was appealed before a county judge who upheld the parents' power to make the decision, in the course of which the case became public and many families offered to take care of the child; however, before the case reached the supreme court, the child died at six days of age [23].

In the case of Ingrid Frank, a German woman who was in a quadriplegic state by a traffic accident, who initially sought rehabilitation but later insisted on being allowed to die; it was provided with a drink containing a cyanide solution that she drank. At the same time, she was filmed, which shows a kind of assisted suicide. For that reason, this is another case that deals with this issue and is important to know as background in the development of euthanasia and assisted suicide [22,23].

## 6 Current and future perspectives

The definition of brain death, the rational use of the concept of euthanasia and assisted suicide, and scientific literacy are the objectives of global bioethics to regulate euthanasia and assisted suicide, which can be accessible in all health systems [[24], [25], [26], [27], [28], [29], [30]]. End-of-life care will continue to be a subject of debate due to the struggle between biomedical principles, the different existing legal frameworks, and the general population's beliefs. Medical education and preparation in the perception of death, especially of a dignified death, seems to be the pillar of the understanding of the need to develop medical-legal tools that guarantee the integrity of humans until the end of their existence [31,32]. This is the reason why the new generations of physicians must be trained in bioethics to face these ethical conflicts during the development of their professional careers.

In addition, although the conception of bioethics belongs to the Western world, it is crucial to take into account the point of view of other cultures and creeds, for example, a study carried out in Turkey, where nursing students were questioned, found that many of them understood the reasons for performing euthanasia; however, they know that Islam prohibits it, as well as its legislation, and therefore they would not participate in this type of procedure [33]. Furthermore, Christianism and Islam prohibit euthanasia, but Judaism also prohibits it; in general, the so-called Abrahamic religions are contrary to any form of assisted death, whether it is active euthanasia, passive, or assisted suicide [34].

## 7 Conclusiones

The history and evolution of euthanasia and assisted suicide have been traumatic throughout human history. The church, politics, and biomedical research have been decisive in defining these concepts. Over the years, the legal framework and bioethical concepts on euthanasia have been strengthened. However, there is still much work to educate the general population and health professionals about end-of-life care and dignified death.

It is also important to remember that life is a concept that goes beyond biology. Currently, bioethics seeks to prioritize the concept of dignity, which must be linked to the very definition of life. Although the phrase is often heard that it is not necessary to move to be alive, what is important is that person feels worthy even if they have limited movement. The person's treatment must be individualized in bioethics since each individual is a unique unit. Therefore, medical paternalism must be abandoned. Instead, the subject must be more involved to understand their context and perception of life and dignity.

## Ethical approval

It is not necessary.

## Sources of funding for your research

None.

## Author contribution

All authors equally contributed to the analysis and writing of the manuscript.

## Provenance and peer review

Not commissioned, externally peer reviewed.

## Research registration Unique Identifying number (UIN)

1. Name of the registry: Not applicable.
2. Unique Identifying number or registration ID: Not applicable.
3. Hyperlink to your specific registration (must be publicly accessible and will be checked): Not applicable.

## Guarantor

Sabrina Rahman. Independent University, Dhaka, Bangladesh. sabrinaemz25@gmail.com.

## Declaration of interests

The authors declare that they have no known competing financial interests or personal relationships that could have appeared to influence the work reported in this paper.
